# Restorative Qualities of and Preference for Natural and Urban Soundscapes

**DOI:** 10.3389/fpsyg.2017.01705

**Published:** 2017-10-04

**Authors:** Paulina Krzywicka, Katarzyna Byrka

**Affiliations:** Wrocław Faculty of Psychology, SWPS University of Social Sciences and Humanities, Wrocław, Poland

**Keywords:** restoration, restorative qualities, sounds, sonic environment, natural soundscapes, urban soundscapes, preference

## Abstract

Psychological restoration in urban agglomerations has become a growing challenge. Although scientific proof of the significance of nature is irrefutable, an increase in built-up areas has led to a decrease in urban greenery. Thus, a growing need for restorativeness in urban surroundings has emerged. To investigate whether positively evaluated sonic environments, represented by natural and urban sounds, have comparable restorative qualities we conducted two studies. The aim of the first (Study 1) was to explore the restorative qualities of positively assessed natural and urban sounds. Participants (*N* = 88) were asked to listen and to rate 22 recordings (each 1 min long) either from natural or urban environments. In the second (Study 2) we investigated whether positively evaluated sonic environments (natural and urban), demand for restoration (feeling relaxed or fatigued) and company (being alone or with a friend) affect the restorative qualities of natural and urban soundscapes. After reading assigned scenarios (feeling relaxed or fatigued; being alone or with a friend), participants (*N* = 120) were asked to imagine a walk in presented sonic environments and to complete forms (one for each sonic environment) concerning the restorative qualities of given soundscapes (natural and urban). Top five recordings of natural and urban sonic environments were selected from Study 1 and combined into a 154-s soundtrack, to provide a background for the imagined walks in both settings. Our findings confirmed that natural sounds are perceived more favorably than urban recordings. Even when only the most positively assessed soundscapes were compared, nature was still perceived as being more restorative than urban areas. Company of a friend was found to be more beneficial in the urban surroundings, particularly when there was no need for restoration.

## Introduction

A recent report of the United Nations (2015) predicts that sixty-six percent of the world population will reside in urban areas by the year 2050. As progressive urbanization seems inevitable, it is essential to seek restorativeness in urban surroundings. Surprisingly, despite researchers’ growing interests in the affordances of various environments, relatively little is known about the restorative qualities of urban areas. Even less explored is the potential of positively assessed urban sounds, which are omnipresent and accompany citizens in their everyday routines.

Research has shown that people’s encounters with natural environments translate into numerous psychological benefits such as improved cognitive functions (Tennessen and Cimprich, 1995; Hartig et al., 2003; Berto, 2005; Berman et al., 2008, 2012; Faber Taylor and Kuo, 2009; Kaplan and Berman, 2010), lessened anger (Cackowski and Nasar, 2003; Kweon et al., 2008), boosted positive affective states (Berman et al., 2008, 2012; van den Berg and Custers, 2011) and reduced stress (Ulrich et al., 1991; Parsons et al., 1998; Wells and Evans, 2003; Kweon et al., 2008; van den Berg et al., 2010; van den Berg and Custers, 2011; Ward Thompson et al., 2012; Hartig et al., 2014).

Urban environments’ positive contributions, meanwhile, have remained obscure, attracting less scholarly attention. Nevertheless, findings from settings such as museums (Kaplan et al., 1993; Packer, 2008), monasteries (Ouellette et al., 2005), or houses of worship (Herzog et al., 2010) have highlighted the positive aspects of man-made surroundings and confirmed that urban environments may also afford mental recovery. In addition, participation in cultural life (e.g., going to the cinema, theater, or concerts) was found to be positively related to perceived health and well-being (Johansson et al., 2001), and attending sporting events and cultural activities was associated with high life satisfaction and good health (Leadbetter and O’Connor, 2013). Due to the fact that well-being and life satisfaction appear to be related to mental recovery (Sonnentag and Fritz, 2015), there is a distinct possibility that urban facilities, in particular those associated with sport and culture, can also contribute to restoration.

Two complementary approaches explain mechanisms behind the restorative effects of environments, the Attention Restoration Theory (ART; Kaplan and Kaplan, 1989; Kaplan, 1995), and the Stress Recovery Theory (SRT; Ulrich, 1983; Ulrich et al., 1991). Although both refer mostly to nature, it has been confirmed that they apply also to the urban surrounding (Kaplan et al., 1993; Ouellette et al., 2005; Herzog et al., 2010).

In line with the former approach (ART) to achieve a restorative effect, an environment should have the ability to reconcile individual desires and goals with environmental demands (‘compatibility’), be perceived as coherent and of substantial scope (‘extent’), create a sense of psychological distance from the problems of everyday life (‘being away’), and induce ‘fascination’ (Kaplan and Kaplan, 1989; Kaplan, 1995). In some built environments additional factors play a role. For example, ‘spirituality’ is characteristic for places that serve religious purposes, such as monasteries (Ouellette et al., 2005) or houses or worship (Herzog et al., 2010), while ‘comfort,’ related to the original property of ‘compatibility’ (Kaplan et al., 1993), defines places such as museums. Other features, meanwhile, may be imperceptible, as was the case with ‘compatibility’ in a study by Herzog et al. (2010). Interestingly, these properties can also be linked. ‘Beauty,’ for example, can be described as a combination of ‘fascination,’ ‘extent,’ and ‘aesthetics’ (Ouellette et al., 2005; Herzog et al., 2010).

The Attention Restoration Theory (Kaplan and Kaplan, 1989; Kaplan, 1995) holds that recovery from mental fatigue occurs through the restoration of depleted cognitive resources. Kaplan (1995) distinguishes two types of attention: stimuli-driven involuntary attention, which does not require mental effort, and directed attention, which is responsible for intentional actions and everyday behaviors. Involuntary attention, due to its bottom-up mechanism (Berman et al., 2008), provides psychological restoration, as attention is automatically captured by fascinating stimuli (Beute, 2014), whereas directed attention requires more conscious control, and when depleted, leads to deterioration of cognitive functions and, subsequently, to a reduction in the ability to adapt to daily demands (Kaplan and Kaplan, 2003).

In line with Stress Recovery Theory (Ulrich, 1983; Ulrich et al., 1991), apart from coping with mental fatigue, restoration can also be explained in terms of the physiological reaction to stress (Ulrich, 1983; Ulrich et al., 1991). Particularly relevant seems the importance of positive affective reactions, which according to Ulrich et al. (1991, p. 224), are “comprised of likening and moderate to high interest, should motivate and sustain prolonged attention/intake, should produce higher levels of positive feelings, reduce negatively toned or stress related feelings such as fear and anger, and suppress stressful or extraneous thoughts (Ulrich, 1981, 1983).”

A vast amount of literature attests to people’s preferences for natural settings. Nonetheless, these findings might be to some extent affected by the selection of urban stimuli. Despite Ulrich’s emphasis on positive affective reaction (Ulrich et al., 1991), in previous works, urban surroundings have often been represented either by unattractive images of industrial zones (Purcell et al., 2001), streets and buildings (Purcell et al., 2001; Joye et al., 2013), which are usually accompanied by automobile traffic (Emfield and Neider, 2014), or by unpleasant audio stimuli such as office noise (Jahncke et al., 2015), sounds of busy intersections, construction site clamor (Medvedev et al., 2015), traffic noise (Gidlöf-Gunnarsson and Öhrström, 2007; Alvarsson et al., 2010), or Time’s Square’s cacophony of conversations, cars, and honks (Emfield and Neider, 2014).

Moreover, conclusions on people’s preference for natural or urban environments have mostly relied on visual stimuli (Herzog et al., 1997; Purcell et al., 2001; Staats et al., 2003; Staats and Hartig, 2004; Nordh et al., 2009; Joye et al., 2013; Stevens, 2014; Conniff and Craig, 2016). Scarce evidence shows, however, that sonic stimuli, such as positively assessed bird songs or sounds of water may also contribute to psychological restoration (Ratcliffe et al., 2013).

As argued by Ulrich (1983), as well as Conniff and Craig (2016), concentrating exclusively on a visual sensory channel limits our understanding of often complex environments, because photography can depict a given setting only fragmentarily (Nordh et al., 2009). Sound, by contrast, can contribute to a more holistic interpretation of a given surrounding, because of its own restorative potential (Alvarsson et al., 2010; Abbott et al., 2016). The impact of the audio stimuli on the assessment of the environment is reflected in the studies investigating a reciprocal relationship between sound and vision. It has been revealed that natural sounds can positively influence perception of images portraying natural or man-made settings (Carles et al., 1999; Hedblom et al., 2014), whereas deterioration in the sonic environment reduces the evaluation of the surroundings (Carles et al., 1999). Likewise, vision can modify the perception of the audio stimuli (Viollon and Lavandier, 1997; as cited in Carles et al., 1999).

Whereas visual and multimodal approaches have been discussed quite frequently, the relationship between positive evaluation of urban sounds and their restorative qualities have been given much less attention. Therefore, the purpose of this paper is to explore the restorative potential of urban soundscapes. In a pair of studies, we want to verify whether restorative qualities of natural and urban sounds are comparable. In Study 1 we aim to explore people’s preference for recorded natural vs. urban sounds and their restorative qualities. The objective of Study 2 is to investigate whether the demand for restoration and company of others play a role in the perception of urban sounds’ restorative qualities.

## Study 1

In line with the abundant existing literature on visual stimuli, we expected that, in general, natural sounds would be evaluated more positively than urban sounds (Hypothesis 1). We also hypothesized that natural sonic environments will be rated as more restorative than urban sonic environments (Hypothesis 2). Additionally, due to the nature of the urban sonic environment, which is usually louder and noisier than nature, we predicted that sensitivity to sounds would play a role in the assessment of the recordings. We expected that the higher the sensitivity to sounds, the less positive the evaluation of the urban sounds would be (Hypothesis 3). We also aimed to select the most positively assessed sounds of both sonic environments.

### Method

#### Participants

Eighty-eight students from the SWPS University of Social Sciences and Humanities (78% women), aged 19–44 years (*M* = 26.74, *SD* = 6.82), were recruited for the study through an advertisement placed in the university’s building and on its website. All gave informed consent after the short introduction to the study. They were rewarded with credit points and had a chance to win a lottery gift worth, on average, 5$. The study was approved by the ethics committee of the authors’ faculty.

#### Materials and Measures

##### Sounds

High-quality (16 bit, 44.1 kHz) samples representing 22 natural environments and 22 urban environments (**Table 1**) were binaurally recorded by a sound recordist. The presence of people was minimized wherever possible while taking these samples so that the focus could be on the environmental sounds. However, there were some exceptions, such as a café, swimming pool, ice rink and parade, where people’s voices could be heard in the background. The audio levels were normalized; each 1-min recording was scaled following Medvedev et al.’s (2015) example to an average sound level of 64 dB SPL, and saved as a WAV file.

**Table 1:** Sounds used in Study 1.

Natural environment
Blackbird in a clearing, Blackcap in the woods, Corncrake at a pond, Crows, Deer in the rut, Forest (boar and birds), Frogs at a pond, Great reed warbler, Howling wolves, Larks and barred warbler, Meadow in the spring (many birds species), Night in the woods (eagle-owls and wind), Nightingale, Ravens, River, Robin at a river, Sea, Seagulls on a windy day, Summer night (crickets and birds), Swarm of insects, Thunderstorm, Wren at a stream

**Urban environment**

Airport (airplane landing), Street (ambulance), Old town (barrel organ), Café, Amusement park (carousel), Church bells, Concert (orchestra tuning and applause), Construction site, Fireworks display, Highway, Ice rink (people skating), Lawn mower, Parade (brass orchestra), Road work (pneumatic hammer), Fire department (siren), Street noise, Subway (empty subway car), Swimming Pool, Traffic jam (with car horns), Train, Video arcade, Wind chimes

##### Evaluation of the sounds

To assess how positive and/or negative each recording was perceived to be, participants used the Evaluative Space Grid (Larsen et al., 2009). The responses were given on a 5 × 5 grid (25 cells) presented on the paper form. On the *x*-axis, ranging from ‘Not at all’ (0) to ‘Extremely’ (4), participants were asked how positive they felt about the stimulus, whereas on the *y*-axis, also ranging from ‘Not at all’ (0) to ‘Extremely’ (4), they were asked how negative they felt about the stimulus. The answers were then averaged. The negative evaluation was not analyzed in this study.

##### Sensitivity to sounds

Participants’ sensitivity to sounds was measured using the Hyperacusis Questionnaire (Khalfa et al., 2002). On this measure, consisting of 14 questions (e.g., ‘Are you particularly sensitive to or bothered by street noise?’), the responses were given on a 5-point scale, ranging form ‘No’ (0) to ‘Yes, a lot’ (4) and then averaged. Three additional questions concerning the experience of being exposed to noise (‘Are you or have you been exposed to noise?’, tolerance to noise (‘Do you tolerate noise less well as compared to a few years ago?’) and eventual hearing problems (‘Have you ever had hearing problems? If so, of what kind?’) were responded to by choosing ‘Yes’ (1) or ‘No’ (0).

According to Khalfa et al. (2002), the mean total score for the questionnaire is 15 ± 6.7, and a result above 28 (out of the maximum score of 42) may be an indicator of auditory hypersensitivity. In our research the Cronbach’s alpha for the scale was 0.76.

##### Restorative qualities of sounds

Each sound’s restorative quality was assessed with four items inspired by the properties of the Attention Restoration Theory (Kaplan and Kaplan, 1989; Kaplan, 1995): being away (‘How often this sonic environment can facilitate a sense of mental detachment from the problems of everyday life?’), fascination (‘How often this sonic environment can induce fascination/interest?’), compatibility (‘How often this sonic environment can encourage you to find yourself in this environment?’) and extent (‘How often this sonic environment can offer many opportunities to act in such area?’). The answers were given on a 5-point scale ranging from: ‘Never’ (1) to ‘Very often’ (5) for questions concerning properties of being away and compatibility, and from ‘Very often’ (1) to ‘Never’ (5) for questions concerning properties of fascination and extent. To ease the calculations, the responses were later recoded (from 0 for ‘Never’ to 4 for ‘Very often’) and averaged. Cronbach’s alpha for the scale was 0.93 (for all natural sounds’ restorative qualities) and 0.84 (for all urban sounds restorative qualities). For the top five sounds of both environments, Cronbach’s alpha was also calculated and was 0.89 (for natural recordings) and 0.81 (for urban recordings). Additionally, we asked participants whether they recognized the presented recording (‘Yes’ or ‘No’) and, if the answer was affirmative, to identify the given sound (an open question).

#### Procedure

Each participant took part in the study individually. After a brief instruction on the procedure, participants were randomly assigned to the natural or urban condition. Subsequently, they listened to and evaluated 22 recordings from natural or urban environments (depending on the condition), played via closed-back headphones (they had the opportunity to adjust the sound volume according to their needs). After each recording, participants rated how positive and negative they felt about the stimulus using the Evaluated Space Grid (Larsen et al., 2009). Then, they were asked whether they recognized the sound and evaluated each sound’s restorative quality. A 22 × 22 Latin square in a Williams design (Latin Squares – Williams Design, n.d.) was used to eliminate order effect. The time needed to complete each questionnaire before the next recording was played was adjusted to each participant’s individual needs. After all recordings were played, participants completed the Hyperacusis Questionnaire (HQ) and basic socio-demographic data. The study lasted around 40 min on average.

### Results

#### Evaluation of All Sonic Environments

We found a significant difference in the evaluation of all recordings, Welch’s *F*(1,55.12) = 23.88, *p* < 0.001, est. ω^2^ = 0.21. As predicted (Hypothesis 1), the natural sounds were perceived more positively (*M* = 2.12, *SD* = 0.65) than the recordings from the urban environments (*M* = 1.55, *SD* = 0.33). In line with expectations (Hypothesis 2), they were also rated as being more restorative (*M* = 8.63, *SD* = 1.44) than urban recordings (*M* = 6.67, *SD* = 1.01), *F*(1,81) = 52.39, *p* < 0.001, η^2^ = 0.39. Further analysis revealed that evaluation of the sounds was positively correlated with their perceived restorative qualities, both for the natural, *r* = 0.78, *p* < 0.001, and urban, *r* = 0.52, *p* < 0.001, sonic environments.

Participants had no major problems identifying the sounds. The most recognizable sounds of the natural environment turned out to be: a blackcap in the woods (100% recognition), crows (100%), howling wolves (100%), insects (100%), the sea (100%), seagulls on a windy day (100%), a thunderstorm (100%), a wren at a stream (100%); the least recognizable were the recordings of a boar (64%) and a deer in the rut (66%). It should be noted that the knowledge of bird species was not required. As for the urban environment, the most recognizable sounds were: an airplane landing (100%), an ambulance (100%), a café (100%), church bells (100%), a fireworks display (100%), a parade (100%), and a traffic jam (100%), whereas the least recognizable were: an ice rink (71%) and a subway (57%). Interestingly, not all who declared sound recognition identified the recordings appropriately (e.g., the deer was taken for marine mammals).

The mean total score for the Hyperacusis Questionnaire (Khalfa et al., 2002) was 15.78 (*SD* = 6.41). Only 3.41% of participants met the criteria of hypersensitivity to sounds (results above 28). Still, positive correlation between participants’ sensitivity to sounds and the positive evaluation of the natural sounds was found, *r* = 0.42, *p* < 0.01. Surprisingly, an increase in sensitivity to sounds turned out to be correlated with a more positive assessment of the natural sounds. Contrary to our expectations (Hypothesis 3), the correlation between sonic urban environments and sensitivity to sounds was non-significant, *r* = -0.20, *p* = 0.19.

#### Most Positively Evaluated Sonic Environments (Natural and Urban)

On the basis of the means of all sounds used in this study (Supplementary Table 1), the five most positively assessed recordings (**Table 2**) from each environment were selected. The chosen recordings in natural settings were: a robin at a river, a wren at a stream, a blackbird in a clearing, the sea, and a blackcap in the woods. The chosen recordings in urban settings were: a concert (an orchestra tuning and applause), a fireworks display, an old town sound (a barrel organ), an amusement park (a carousel), and a café.

**Table 2:** The most positively assessed sounds of natural and urban environments in Study 1 (mean values, standard deviations).

Natural environment	*M*	*SD*	Urban environment	*M*	*SD*
Robin at a river	3.48	0.82	Concert	3.59	0.62
Wren at a stream	3.43	0.93	Fireworks display	2.95	1.06
Blackbird in a clearing	3.14	1.00	Old town	2.80	0.95
Sea	3.09	0.88	Amusement park	2.77	0.99
Blackcap in the woods	3.02	1.05	Café	2.77	1.16

A significant difference was found between the evaluations of the sonic environments, *U* = 640.00, *p* < 0.01. Again, natural sounds were perceived more positively (*Mdn* = 3.40) than urban recordings (*Mdn* = 3.00). Furthermore, natural sonic environments were rated as being more restorative (*M* = 12.12, *SD* = 1.90) than urban recordings (*M* = 11.17, *SD* = 1.82), *F*(1,86) = 5.71, *p* < 0.05, η^2^ = 0.06. As was previously discovered, the evaluation of the sounds was found to be correlated with perceived restorative qualities of the top five natural sounds, *r*_s_ = 0.80, *p* < 0.001, and for the top five urban recordings, *r*_s_ = 0.61, *p* < 0.001.

The relationship between a participant’s sensitivity to sounds and the evaluations of the sonic environments was also examined. Neither the correlation between auditory sensitivity and natural sound assessments (*r*_s_ = 0.28, *p* = 0.06) nor the correlation between auditory sensitivity and urban sound assessments (*r*_s_ = -0.19, *p* = 0.23) was significant, possibly suggesting that sensitivity to sounds did not influence the evaluations of positive sonic environments.

### Discussion

As expected, our study confirmed that natural environments, represented by sound stimuli, are perceived more favorably than urban surroundings. This is quite intuitive, as it could have been predicted that negatively perceived natural sounds, such as a crow’s call (Ratcliffe et al., 2013), are still less disturbing than some intrusive urban sounds (e.g., recording of a pneumatic hammer). We found, however, that even positive assessment of the urban sounds cannot equalize the difference in how both environments are perceived.

The high percentage of sound identification shows that both sonic environments were familiar to participants, so there may be a different explanation of these results, other than distinct familiarity with the soundscapes. It is possible, for example, that people find urban surroundings more heterogeneous than nature and that participants’ individual preferences toward the given settings (even when sonic) are reflected in the study. Our findings seem to confirm that. Whereas natural sounds were almost unanimously assessed positively (in particular birdsongs and water-related soundscapes), the ratings of urban recordings varied. It is likely that some participants found the sonic atmosphere of the urban area as a model example of the environments conducive to recreation and social life, while others described the same setting as undesirable: fatiguing, too noisy, or even aesthetically unappealing.

In line with our assumptions, natural recordings were also perceived as being more restorative than urban soundscapes, both for all and for the top five sounds from natural and urban environments. It was also confirmed that evaluations of the sounds were positively correlated with their perceived restorative qualities, especially in the natural sonic environment.

Surprisingly, sensitivity to sounds did not affect the perception of urban sounds, neither for all recordings nor for the top five. It did, however, influence the assessment of all natural sounds. These results may indicate that, despite preferring natural recordings, people who are sensitive to sounds has become highly accustomed to urban soundscapes. The most positively assessed sounds, meanwhile, are not intrusive, so their origin does not matter.

To further explore the relationship between positive evaluation and restorativeness, we decided to conduct another study, in which we wanted to more thoroughly examine soundscapes’ restorative qualities. Due to the fact that sensitivity to sounds did not turn out to significantly influence the perception of urban soundscapes and considering that we had already selected the most positively assessed sounds of both sonic environments (taking sensitivity to sounds into account), we decided to exclude sensitivity to sounds from further research. Instead we decided to concentrate on additional factors that can influence soundscapes’ restorative qualities – company and the demand for restoration.

## Study 2

In Study 2, we further investigated whether positively evaluated urban surroundings can afford psychological restoration comparable to natural settings. For this purpose we replicated the study by Staats and Hartig (2004), in which participants were asked to imagine themselves in a given environment during an imagined walk with a friend or alone. Instead of visual presentation of environments, we used positively assessed sound stimuli.

We were interested in whether a presence in a given setting, even when imagined, can affect its perceived restorative quality. As a sonic background for the imagined walk we chose the most positively evaluated recordings from Study 1. Based on the results from Study 1, we expected that there would be a significant difference between natural and urban soundscapes’ restorative qualities (Hypothesis 1).

Additionally, inspired by Staats and Hartig (2004), we decided to verify how company and the demand for restoration (feeling attentionally fatigued or relaxed) interplay with positively assessed soundscapes from natural and urban environments. We expected that participants would experience more restoration being in company, both in the natural (Hypothesis 2a) and in the urban (Hypothesis 2b) soundscapes. We also predicted that demand for restoration would affect the evaluation of restorative qualities: it was expected that participants from the attentionally fatigued condition would rate an imagined walk in the natural soundscapes as being more restorative than an imagined walk in the urban sonic environment (Hypothesis 3a), whereas participants from the relaxed condition, due to the use of stimuli representing positively assessed soundscapes, would rate an imagined walk in the urban soundscapes as being more restorative than an imagined walk in the natural sonic environment (Hypothesis 3b). It was also predicted that there would be a difference in the evaluation of the imagined walk in the natural and the urban soundscapes (Hypothesis 4).

In addition, we wanted to determine whether and how a sense of security influences the restorative qualities of positively perceived sonic environments. We assumed that the urban soundscapes would invoke a greater sense of security than the natural soundscapes (Hypothesis 5). It was also expected that those who imagine themselves walking with a friend would feel more secure than those who imagine themselves walking in solitude, both in the natural (Hypothesis 6a) and the urban (Hypothesis 6b) soundscapes. Moreover, we predicted that a sense of security would have an indirect effect on restorative qualities both in the natural (Hypothesis 7a) and the urban (Hypothesis 7b) sonic environments through company. Likewise, we expected that a positive evaluation of an imagined walk would have an indirect effect on restorative qualities in the natural (Hypothesis 8a) and the urban (Hypothesis 8b) soundscapes through company.

### Method

#### Participants

One hundred and twenty participants (78% women) aged 18 to 51 years (*M* = 25.41, *SD* = 8.38) took part in the study. All were students of the SWPS University of Social Sciences and Humanities who responded positively to the announcement about this research that was placed in the university’s building and on its website. Each participant signed the informed consent form that outlined the procedure’s steps, and was rewarded with credit points and a chance to win a lottery gift worth, on average, 5$. The study was approved by the ethics committee of the authors’ faculty.

#### Materials and Measures

##### Sound recordings

The most-positively assessed sounds from Study 1 (**Table 2**) were used: five natural (a robin at a river, a wren at a stream, a blackbird in a clearing, the sea, and a blackcap in the woods), and five urban recordings (a concert, a fireworks display, a barrel organ, a carousel, and a café). However, this time, to facilitate the listening procedure, each sound was shortened to 30 s, as advised by participants of Study 1, and then combined with others (with 1-s transitions) into a single 154-s soundtrack to provide a background for the imagined walks in the natural and urban settings. Each sound in the compilation represented one place on the 1-h walking route, which, depending on the participant’s imagination, could have been either visited or just passed by.

##### Scenarios

Questionnaires and scenarios used in this study were based on translations of the items and scenarios described by Staats and Hartig (2004).

*Demand for restoration scenario.* Two alternative scenarios of demand for restoration were created. In a random manner, participants were asked either to imagine themselves being attentionally fatigued (one half of participants) or completely relaxed (the other half). In the former, participants were instructed to visualize a state of tiredness at the end of a semester, when problems with concentration and negative emotions occur. In the latter, participants were instructed to imagine a state of relaxation after the summer holidays, when feelings of restoration, energy, and preparedness to concentrate on learning occur. The demand for restoration scenario remained unchanged throughout the course of the study.

*Company scenario.* Two versions of a story were prepared. In both, participants were asked to imagine themselves strolling for an hour in the environment, which was represented by the sounds they were about to hear (natural or urban). In one version, the participants were instructed to imagine a lonely walk, without anyone they knew. In the other, they were to imagine walking with someone close to them, for example, with a friend. Again, participants were randomly assigned to one of these two scenarios, and the scenario remained unchanged throughout the study.

##### Manipulation checks

Manipulation checks consisted of eight items measuring affective state (feeling irritated, tired, exhausted, mentally drained) and attention depletion (ability to make a decision, concentrate, predict consequences of a complex situation, focus attention during a long lecture). Participants indicated to what extent they agreed or disagreed with each sentence using a scale from ‘Completely not’ (1) to ‘Very much’ (7). The scale for attention depletion was reversed, and the mean across all eight items was calculated. The Cronbach’s alpha for manipulation checks was 0.93.

##### Restorative qualities of the imagined soundwalks

Participants rated the restorative qualities of an hour-long walk in sonic environments (natural and urban), assuming they were either attentionally fatigued or relaxed. They were asked 19 questions, based on the work of Staats et al. (2003), about the benefits of an hour-long walk in the given soundscapes. The items concentrated on psychological restoration (e.g., ‘Experience of restoration’ or ‘Regain an ability to concentrate’), reflection (e.g., ‘Thinking about important matters’ or ‘View things from a new perspective’), and social stimulation (e.g., ‘Seeing many different people’ or ‘Noticing many different things surrounding you’). The answers were measured on a Likert-type scale ranging from ‘Most unlikely’ (1) to ‘Extremely likely’ (7) and then averaged. Cronbach’s alpha was similar: 0.87 for natural soundscapes, and 0.88 for urban soundscapes.

Additionally, a similar set of items was used to assess the pleasantness of an imagined walk, with answers ranging from ‘Very unpleasant’ (1) to ‘Very pleasant’ (7), however, the results were not analyzed in this study.

##### Sense of security

Participants’ sense of security in natural and urban soundscapes was measured. They rated how secure they felt in the given sonic environment on a 7-point scale from ‘Completely not’ (1) to ‘To a very high degree’ (7).

##### Evaluation of the imagined soundwalks

The imagined soundwalks were evaluated using additional rating instruments. Participants were asked to indicate how attractive, nice, positive, and enjoyable an hour-long walk in a given sonic environment would be. Each of the four answers was given on a Likert-type scale ranging from ‘Completely not’ (1) to ‘To a very high degree’(7). The means were calculated separately for both soundscapes. Cronbach’s alpha was, respectively, 0.87 for the evaluation of the walk in natural soundscapes, and 0.89 for the evaluation of the walk in urban soundscapes.

##### Control measures

For each sonic environment, two additional questions were asked: one about the familiarity of the soundscapes and one about the representativeness of its positively assessed sounds. Answers were given on a Likert-type scale ranging from ‘Not at all’ (1) to ‘Very’ (5). Additionally, the question about familiarity of the state of attentional fatigue or relaxation (depending on condition) was added. This answer was also given on a 5-point scale ranging from ‘Not at all’ (1) to ‘Very’ (5). An open question for comments and opinions about the research was included. Demographic data such as age and gender were also collected.

#### Procedure

We followed the procedure used by Staats and Hartig (2004), with the exception that the photographic slides illustrating classic natural and urban settings (e.g., forest scenes, shopping streets) were replaced with audio stimuli, representing only positively assessed sonic environments from Study 1.

**FIGURE 1:**
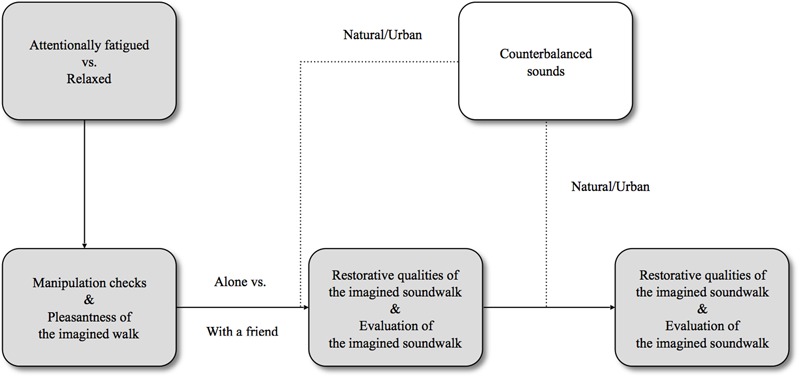
An outline of the research procedure.

Participants, randomly assigned to conditions (*n* = 30 per group), were seated in the middle of a room in front of a pair of sound speakers; individually or in small groups (up to 6 people). After detailed instruction, they read about the demand for a restoration scenario of feeling relaxed or attentionally fatigued and completed the first questionnaire composed of manipulation checks and items measuring pleasantness of an imagined hour-long walk. Next, they read another short text – a company scenario of being alone or with a friend and the first group of environmental sounds (natural or urban) were played, the order of which was balanced: one half of participants heard the set of urban recordings, while the other half heard the set of natural sounds. Each set of soundscapes was presented twice: the first time when the participants were to imagine themselves passing five different locations during an imagined hour-long walk according to prior scenarios, and the second time to refresh their memories and to accompany the questionnaires about the evaluation of the imagined walks and their restorative qualities. The whole procedure was repeated with the other set of sounds, and another questionnaire with identical questions was used. The overall session time averaged 30 min. The scheme of the procedure used in the study is presented in **Figure 1**.

### Results

In this section we first present the results of manipulation checks and preliminary analyses. Then we report the effects of the restorative qualities of the imagined soundwalks. Later in the text we discuss the evaluation of the imagined soundwalks and the influence of sense of security on the obtained results.

#### Manipulation Checks and Preliminary Analyses

We verified whether participants indeed met the given condition of demand for restoration after reading the assigned scenarios. As assumed, those instructed to imagine themselves in a state of attentional fatigue indicated being more fatigued (*M* = 4.87, *SD* = 0.80) than those instructed to imagine themselves relaxed (*M* = 2.16, *SD* = 0.69), *t*(118) = -19.91, *p* < 0.001. The difference in familiarity between the conditions was not significant, *t*(118) = -1.30, *p* = 0.20: participants were familiar with their assigned states of being attentionally fatigued (*M* = 4.50, *SD* = 0.73) or relaxed (*M* = 4.33, *SD* = 0.68).

The natural sonic environment was perceived as more familiar to participants (*M* = 4.63, *SD* = 0.71) than the urban sonic environment (*M* = 4.28, *SD* = 0.90), *t*(119) = 3.39, *p* < 0.01, and recordings of the positively assessed natural soundscapes were perceived as more representative (*M* = 4.74, *SD* = 0.51) than those representing positively evaluated urban soundscapes (*M* = 3.76, *SD* = 0.96), *t*(119) = -10.55, *p* < 0.001.

#### Effects of the Restorative Qualities of the Imagined Soundwalks

A 2 × 2 × 2 mixed ANOVA was carried out to analyze the effects of the restorative qualities of the imagined soundwalks with the type of soundscapes (natural vs. urban) as a within-subjects variable and company (alone vs. with a friend) and demand for restoration (feeling attentionally fatigued vs. relaxed) as between-subjects variables.

As assumed (Hypothesis 1), the results yielded a significant main effect of soundscapes’ restorative qualities, *F*(1,116) = 84.37, *p* < 0.001, $\eta_{p}^{2}$ = 0.42 revealing that participants preferred an imagined walk in the natural soundscapes (*M* = 5.38, *SD* = 0.68) over an imagined walk in the urban soundscapes (*M* = 4.61, *SD* = 0.82).

The main effect of company was statistically significant, *F*(1,116) = 5.56, *p* < 0.05, $\eta_{p}^{2}$ = 0.05. Participants rated sonic environments as being more restorative when they imagined themselves in the company of a friend. However, simple effects analysis revealed that the difference between being in company (*M* = 4.77, *SD* = 0.74) or alone (*M* = 4.44, *SD* = 0.87) was significant only for the urban soundscapes, as predicted by Hypothesis 2b, *t*(118) = 2.24, *p* < 0.05. Contrary to our expectations concerning the natural soundscapes (Hypothesis 2a), the difference between being with a friend or alone was insignificant, *t*(118) = 1.34, *p* = 0.18.

The main effect of the demand for restoration condition (being attentionally fatigued or relaxed) was not statistically significant *F*(1,116) = 0.91, *p* = 0.34 nor was the interaction between company and demand for restoration, *F*(1,116) = 0.88, *p* = 0.35.

The interaction between demand for restoration (feeling attentionally fatigued or relaxed) and the restorative qualities of sonic environments was non-significant, marginally above conventional p-level, *F*(1,116) = 3.69, *p* = 0.057. Contrary to our assumptions, demand for restoration did not influence the restorative qualities for neither attentionally fatigued (Hypothesis 3a) nor relaxed participants (Hypothesis 3b).

**FIGURE 2:**
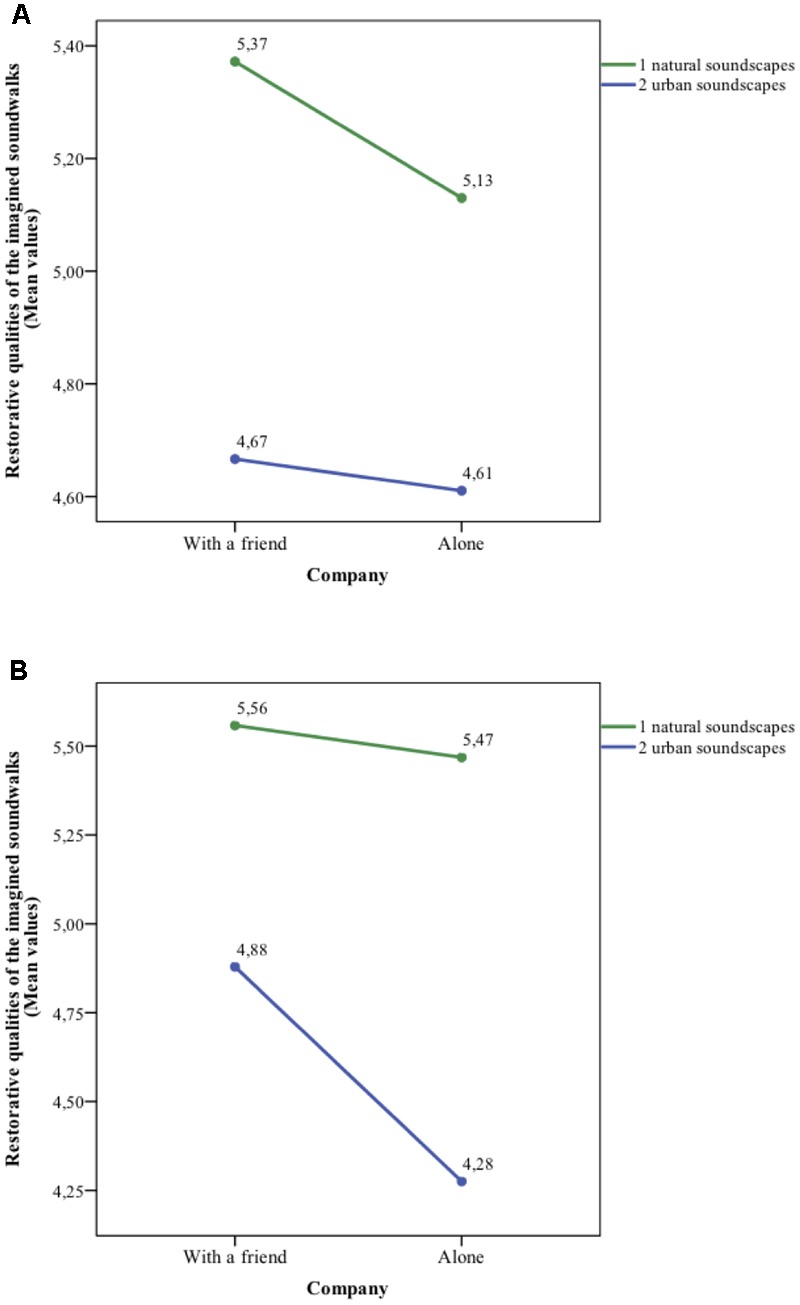
Three-way interaction between the demand for restoration, company and restorative qualities of the imagined soundwalks for attentionally fatigued **(A)**, and relaxed **(B)** participants.

Still, a significant three-way interaction (**Figure 2**) was found between the demand for restoration, company, and the restorative qualities of sonic environments, *F*(1,116) = 4.31, *p* < 0.05, $\eta_{p}^{2}$ = 0.04.

Relaxed participants preferred company over being alone both in the natural (*M* = 5.56, *SD* = 0.54 vs. *M* = 5.47, *SD* = 0.63) and in the urban soundscapes, (*M* = 4.88, *SD* = 0.69 vs. *M* = 4.28, *SD* = 0.82). However, the difference between being alone or with a friend was significant only for the urban sounds, *t*(58) = 3.09, *p* < 0.01. Attentionally fatigued participants favored company over solitude, but the differences were not significant for neither the natural soundscapes, *t*(58) = 1.30, *p* = 0.21 nor the urban soundscapes, *t*(58) = 0.26, *p* = 0.80.

#### Evaluation of the Imagined Soundwalks

As predicted in Hypothesis 4, we found a significant difference in the evaluation of the imagined soundwalks, *Z* = -8.47, *p* < 0.001. Participants preferred the walk in the natural soundscapes (*Mdn* = 6.50) over the walk in the urban soundscapes (*Mdn* = 5.00).

#### Sense of Security

Participants imagining their walks in urban soundscapes were hypothesized to report a greater sense of security than those imagining their walks in natural soundscapes (Hypothesis 5). A significant difference between the two was found, *Z* = -6.90, *p* < 0.001, but in the opposite manner: those imagining walks in a natural sonic environment felt more secure (*Mdn* = 6.00 vs. *Mdn* = 5.00).

Additionally, participants were assumed to feel more secure in company no matter the sonic environments. According to our expectations (Hypothesis 6a), a significant difference in the assessment of security during the imaginary walk in natural soundscapes was found between those assigned to imagine company (*Mdn* = 7.00) and those assigned to imagine solitary walks (*Mdn* = 6.00), *U* = 1302.00, *p* < 0.01. However, contrary to our predictions (Hypothesis 6b), the appraisal of security in the urban soundscapes was the same (*Mdn* = 5.00) regardless of the company scenario, *U* = 1574.50, *p* = 0.22.

#### Effect of Company on Restorative Qualities as a Function of a Sense of Security

We examined two mediation models, one for the natural soundscapes (Hypothesis 7a) and one for the urban soundscapes (Hypothesis 7b), in which the relationship between company and the restorative qualities of sonic environments was mediated by a sense of security.

The standardized regression coefficient between company and a sense of security in the natural soundscapes was statistically significant, *b* = -0.62, *SE* = 0.21, *p* < 0.01, as was the standardized regression coefficient between a sense of security in the natural soundscapes and the restorative qualities of the natural sonic environment, *b* = 0.17, *SE* = 0.05, *p* < 0.01. Because two crucial assumptions of mediation were met (Kenny et al., 1998), further analyses were conducted: a bootstrapped estimation with 5000 samples yielded a significant indirect effect of company on natural soundscapes’ restorative qualities through a sense of security in the natural sonic environment, *b* = -0.10, *SE* = 0.04, 95% CI [-0.22, -0.03]. The mediator accounted for more than half of the total effect, *P*_M_ = 0.62. Company was not a significant predictor of restorative qualities of the natural soundscapes after introducing the mediator to the model, *b* = -0.06, *SE* = 0.12, *p* = 0.61. As predicted (Hypothesis 7a), the relationship between company and natural soundscapes’ restorative qualities was mediated by a sense of security.

Conversely, for the urban soundscapes, company and a sense of security were not significantly related (*b* = -0.31, *SE* = 0.26, *p* = 0.22). Since the mediation assumptions were not met, further tests were not conducted (Baron and Kenny, 1986). Contrary to our expectations (Hypothesis 7b), the mediating effect of a sense of security in the urban sonic environment on the relationship between company and urban soundscapes’ restorative qualities was not confirmed.

#### Effect of Company on Restorative Qualities as a Function of a Positive Evaluation of the Imagined Soundwalks

Because we were interested in the effect of a positive assessment on the perception of sounds, we also wanted to analyze the influence of a positive evaluation of the imagined walk in a given sonic environment (as an individual component extracted from the general evaluation of the imagined walk) on obtained results. For this reason, we tested two models, again one for the natural (Hypothesis 8a) and one for the urban soundscapes (Hypothesis 8b), in which the relationship between company and soundscapes’ restorative qualities was mediated by a positive evaluation of the imagined walk in a given sonic environment.

For the natural soundscapes, the standardized regression coefficient between company and a positive evaluation of the imagined walk in the natural sonic environment was not statistically significant, *b* = -0.18, *SE* = 0.14, *p* = 0.18. As one of the basic mediation assumptions was not met (Baron and Kenny, 1986), further analyses were not conducted. Contrary to our predictions (Hypothesis 8a), the mediating effect of a positive evaluation of the imagined walk in the natural sonic environment on the relationship between company and natural soundscapes’ restorative qualities was not confirmed.

By contrast, for the urban soundscapes, the standardized regression coefficient between company and urban soundscapes’ restorative qualities was statistically significant, *b* = -0.60, *SE* = 0.22, *p* < 0.01, as was the standardized regression coefficient between a positive evaluation of the imagined walk in the urban soundscapes and the urban soundscapes’ restorative qualities, *b* = 0.34, *SE* = 0.05, *p* < 0.001. A bootstrapped estimation with 5000 samples yielded a significant indirect effect of company on the restorative qualities of the urban sonic environment through a positive evaluation of the imagined walk in the urban soundscapes, *b* = -0.21, *SE* = 0.08, 95% CI [-0.38, -0.07]. The mediator could account for more than half of the total effect, *P*_M_ = 0.62. Company was no longer a significant predictor of urban soundscapes’ restorative qualities, *b* = -0.12, *SE* = 0.13, *p* = 0.35. As expected (Hypothesis 8b), the relationship between company and urban soundscapes’ restorative qualities was mediated by a positive evaluation of the imagined walk in the urban sonic environment.

### Discussion

The main purpose of this study was to explore whether positively assessed urban soundscapes have comparable restorative qualities to natural sounds. We found that both positively assessed sonic environments were perceived as having different restorative qualities. An imaginary walk in the natural soundscapes, including birdsongs and water sounds, was evaluated as being more restorative than an imagined walk in the urban sonic environment, represented by sounds from places such as an old town or an amusement park. These results confirm previous findings about the restorative qualities of natural soundscapes, underlining the importance of greenery in urban surroundings.

It appears that the imagined walk did not change the perception of the urban sonic environment, which was still regarded as being less restorative than nature. It is possible that people do not imagine walking in an urban setting to be as restorative as walking in a natural surrounding. They walk in the city every day, commuting to work or school, often with a purpose and in a hurry. Daily routines are rarely associated with pleasure. A stroll in nature, however, could be perceived as something extraordinary, as a ‘being away’ experience with no need to rush and with the aim to relax.

The advantage of a natural sonic environment was also confirmed by a more positive evaluation of the imagined walk in nature. It is likely that perceived familiarity and representativeness of sounds affected these findings. We found that the most positively assessed natural recordings were perceived as more familiar and more representative of a given surrounding than the top five urban sounds. These results can be explained by the notion of comfort with the presented type of setting, described previously by Kaplan et al. (1993).

In Study 2, we also explored the roles of the demand for restoration and company. Participants evaluated restorative qualities of sonic environments more favorably when they were assigned to a company condition; however, the result was significant only for the urban soundscapes. Contrary to our expectations, demand for restoration did not influence perceived soundscapes’ restorative qualities. Although we suspected that urban sounds could evoke hard fascination not conducive to restoration, this was not confirmed in the obtained results.

In an interaction between restorative qualities of sonic environments, company, and demand for restoration, relaxed participants generally favored being in company, but the difference was significant only in the urban soundscapes; attentionally fatigued participants also preferred company, but the differences between being alone or with a friend were not significant for both sonic environments. These findings suggest that the presence of a close friend is more important when one is in the urban setting.

It is probable that the sample of sounds used in the study partially accounted for the results. Most recordings represented urban environments conducive to meetings (e.g., a café) and visits by relaxed people (e.g., an amusement park). Our findings can therefore highlight the social aspect of an urban environment. Because the urban sounds were associated by participants of Study 1 with fun and social interaction, we can assume that participants preferred to share their experience with someone. It seems that loneliness in the urban environment is more disturbing (e.g., a few prefer be alone in a café). The importance of having company in the urban setting, represented by the urban soundscapes, was reflected in mediation analysis. It turned out that a positive evaluation of the imagined walk in the urban sonic environment mediated the relationship between company and urban soundscapes’ restorative qualities.

Company also increased the sense of security, but we found an interesting contradiction here. Although the urban soundscapes were perceived as less secure, it made no difference to participants whether they had company or not in urban sonic environment, but in the natural soundscapes, perceived as more secure, they preferred to be with someone. Evidently, company of others plays a different role in natural than in urban environments.

In consonance with previous research, in which visual stimuli were used (Staats and Hartig, 2004), mediation analysis confirmed the indirect effect of a sense of security on the relationship between company and the restorative qualities of natural sonic environment. These results might suggest that the urban environment, portrayed by urban sounds, is more predictable (participants know what to expect from the setting) and therefore participants are more likely to explore it alone.

## General Discussion

The combined findings from both studies suggest that natural sounds are perceived by people as being more restorative than urban recordings. This result holds even if only positively assessed urban sounds are used, and when additional factors, such as company or demand for restoration, are considered.

A possible explanation of these findings, which we have not discussed yet, is that natural and urban sounds differ in the extent to which they involve soft or hard fascination. According to Kaplan (1995), these two types of fascination coexist. Soft fascination, characterized by moderate intensity, is experienced in aesthetically pleasant environments, whereas hard fascination is much more intense and appears in more attentionally demanding environments (Heintzman, 2002), such as an exciting NBA match (Chow and Lau, 2015). In our study, natural sounds could have supported restoration by inducing soft fascination in contrast to urban sounds evoking hard fascination. While soft fascination restores concentration, and thus contributes to restoration to a greater extent than hard fascination (Reese and Myers, 2012), it is possible that our findings repeated this pattern. However, it seems that hard and soft fascination can also co-occur. In Study 1, for example, a concert recording, which was the most positively assessed sound of both environments, could have simultaneously induced hard fascination, because of its complexity, and soft fascination, because music is known for its ability to facilitate calm, tranquility and well-being (Krout, 2007; Labbé et al., 2007; Laukka, 2007; Linnemann et al., 2015).

The preference for natural soundscapes might also be explained by the psycho-evolutionary approach. It is believed that “for most of the millions of years during which our species evolved, humans coexisted in a close relationship with the natural environment. Therefore, most adaptations in the human organism, including those of the brain and related behavioral reactions, developed as an evolutionary response to needs imposed by this environment. In contrast, the history of human civilization is relatively short” (Hartig et al., 2011, p. 142). Hence, it is possible that despite the positive valence of urban areas represented by sounds associated with pleasant leisure activities, such as a concert, participants could nonetheless express an innate preference for nature represented by birdsongs and water-related recordings. Notably, because positively assessed urban sounds were mainly linked to recreation and entertainment, these results show that an urban soundscape cannot be equated to noise and its positive aspect should be more broadly acknowledged.

A few limitations of our studies should be mentioned. Because we wanted to depict different locations and use stimuli often used in other research, some of the sounds chosen for Study 1 could have been rated ambivalently or negatively (e.g., crows or road work), thus limiting the selection of potentially positive sounds. As a result, even though we made every effort to provide a variety of sounds, some of the urban facilities or natural habitats, which could have been assessed positively, were not represented (e.g., sports arena).

Moreover, the results obtained in Study 2 could have been partly affected by the role that imagined walking imposed on participants. Although in real life walking in natural surroundings (without additional activities) is a common form of recreation, in urban settings, especially ones similar to those presented in the study (e.g., an amusement park or café), the walk could be additionally associated with other activities accessible in such environments. Thus, for instance, the presence in an amusement park could be related to partaking in the entertainment offered in such places (e.g., taking a carousel ride), because it is rather unlikely to visit such a place just to stroll between attractions. As a result, the predetermined role of ‘an observer’ instead of the role of ‘an active participant’ could have resulted in a less positive evaluation of the restorative qualities of the urban soundscapes.

It should also be noted that our findings are limited to one modality (auditory), which may deprive us of some potentially relevant data. Therefore, in the future we would like to take a multimodal approach, which better mimics real-life experience.

In further research on restorative qualities of natural and urban environments, participants should be allowed to perform an attention-exhausting task prior to the beginning of the study. Future research could also investigate whether the presence of a real friend affects the results and whether differences in responses can be found between participants tested individually or in a group.

It is also worthwhile to mention the procedure used in Study 2. We based our scenarios and items on the ones described by Staats and Hartig (2004); however, it would be interesting to modify them, exploring in detail the impact of the evaluation of the environment on its restorative potential or personal preferences for activities and places associated with regeneration. Furthermore, items measuring restorative qualities of a given soundscape/surrounding could also include questions derived from other discovered factors of the ART, such as comfort with a given type of setting (Kaplan et al., 1993) or beauty (Ouellette et al., 2005; Herzog et al., 2010).

Additionally, individual differences between participants should be taken into account. The results found by Meagher (2016) suggest that not only the type of environment, but also personality traits, such as neuroticism, can influence psychological restoration. Future research should also examine the effects of stereotypes on the perception of the natural and urban sonic environments/settings and focus on the restorative qualities of urban surroundings.

Moreover, because a healthier urban biosphere, in which a place for coexistence of natural and artificial worlds would be found, seems to be potentially beneficial for urban residents, it is worthwhile to search for new, effective solutions that could have been implemented on macro (e.g., the WHO Healthy Cities project^1^) and micro scales (e.g., pocket gardens), to promote mental and physical heath within the urban surrounding. The idea that soundscapes, such as the murmur of a stream or birdsong, may change the perception of a given urban environment seems very appealing, as we have not yet fully discovered restorative qualities of sonic environments. Therefore, it would be interesting to further explore the way in which the relationship between sensory modalities (more than one) affects an environment’s restorative qualities in a positive (what increases it) or negative (what decreases it) direction. It would also be worthwhile to concentrate more on the mutual penetration between natural and urban environments instead of viewing them as opposites.

## Conclusion

In sum, our studies provided new knowledge about positively assessed urban sounds and their impact on the perceived restorative qualities of urban areas. Although the obtained results do not favor urban sonic environment, they emphasize the importance of natural soundscapes within an urban surrounding and the role of well-planed urbanized areas, which, with balanced artificial and natural settings, can be more beneficial to urban residents by promoting well-being, improving perceived quality of life and creating new spaces with potential for restoration within urban agglomerations.

## Ethics Statement

These studies were carried out in accordance with the recommendations of the ethical guidelines of the faculty’s ethics board with written informed consent from all subjects. All subjects gave written informed consent in accordance with the Declaration of Helsinki.

## Author Contributions

Research idea and study design: PK and KB. Data collection: PK. Data analysis and paper writing: PK and KB.

## Conflict of Interest Statement

The authors declare that the research was conducted in the absence of any commercial or financial relationships that could be construed as a potential conflict of interest.

## Footnotes

1 For more information about the WHO Healthy Cities project, see de Leeuw (2001).
