# Supplementary material for: Restorative Qualities of and Preference for Natural and Urban Soundscapes
Source: Front Psychol. 2017 Oct 4;8:1705. doi: 10.3389/fpsyg.2017.01705 (PMC5632731; doi:10.3389/fpsyg.2017.01705)
Supplement: Supplementary file 1 [file Table_1.pdf]

Supplementary Table 1. Mean values and standard deviations of sounds from natural and urban environments

**Natural environment**	***M***	***SD***	**Urban environment**	***M***	***SD***
Robin & river	3.48	0.82	Concert	3.59	0.62
Wren & stream	3.43	0.93	Fireworks	2.95	1.06
Blackbird & clearing	3.14	1.00	Barrel organ	2.80	0.95
Sea	3.09	0.88	Carrousel	2.77	0.99
Blackcap & wood	3.02	1.05	Café	2.77	1.16
Larks & barrel warbler	2.89	1.13	Parade (brass orchestra)	2.75	1.06
River	2.72	1.10	Swimming pool	2.48	1.15
Nightingale	2.50	1.25	Wind chimes	2.30	1.17
Meadow (many birds)	2.39	1.21	Church bells	2.16	1.18
Great reed warbler	2.32	1.01	Airplane landing	1.93	1.13
Seagulls & wind	2.20	1.39	Ice rink	1.55	1.17
Night (crickets & birds)	2.00	1.10	Video arcade	1.27	1.26
Ravens	1.98	1.09	Highway	1.05	1.06
Thunderstorm	1.80	1.44	Lawn mower	0.82	1.06
Frogs	1.73	1.19	Empty subway car	0.64	0.94
Forest (boar & birds)	1.45	1.21	Street noise	0.55	0.98
Crows	1.41	1.15	Train	0.50	0.76
Corncrake	1.32	1.18	Street (ambulance)	0.41	1.09
Eagle-owls	1.18	1.23	Construction site	0.27	0.62
Deer in the rut	1.05	1.22	Fire department (siren)	0.23	0.74
Howling wolves	0.43	0.97	Pneumatic hammer	0.20	0.55
Swarm of insects	0.41	0.69	Traffic jam & car horns	0.18	0.39
